# EATME: An R package for EWMA control charts with adjustments of measurement error

**DOI:** 10.1371/journal.pone.0308828

**Published:** 2024-10-03

**Authors:** Li-Pang Chen, Cheng-Kuan Lin

**Affiliations:** Department of Statistics, National Chengchi University, Taipei, Taiwan, ROC; University of the Punjab, PAKISTAN

## Abstract

In this paper, we introduce an R package **EATME**, which is known as Exponentially weighted moving average (EWMA) control chart with Adjustments To Measurement Error. The main purpose of this package is to correct for measurement error effects in continuous or binary random variables and develop the corrected control charts based on the EWMA statistic. In addition, the corrected control charts can detect out-of-control process accurately. The package contains a function to generate synthetic data and includes functions to determine the reasonable coefficient of control limit as well as estimate average run length. Moreover, for the visualization, we also provide the control charts to show the monitoring of in-control and out-of-control process. Finally, the functions in this package are clearly demonstrated, and numerical studies show the validity of the package.

## 1 Introduction

In the field of industrial statistics, statistical process control (SPC) has been an important topic, which aims to monitor productions of products and detect potentially out-of-control (OC) process. The main purpose is to develop valid control charts for the production monitoring, so that the reliability in production lines is ensured.

A typical dataset includes continuous random variables or binary random variables, where the latter structure reflects (non-)conforming products. To analyze the continuous random variables, one may focus on location process or dispersion process, and several control charts have been applied, such as parametric settings for Shewhart charts, exponentially weighted moving average (EWMA) charts, and cumulative sum (CUSUM) charts summarized by [1], EWMA mean charts [2], variability monitoring (e.g., [3]), likelihood ratio-based EWMA control charts [4], and nonparametric methods [5]. On the other hand, to deal with the binary random variables, the *p*-control chart [1] is perhaps a commonly used approach, and relevant extensions have been developed, such as quasi average run length (ARL)-unbiased *p*-control charts based on a heuristic method [6], the risk adjustment control charts for categorical random variables [7], and the general framework of distribution free settings [8].

However, noisy data usually inevitably exist when collecting the data. One of typical structures of noisy data is *measurement error*, which is occurred due to imprecise machine or wrong records caused by investigators. As commented by [9], ignoring measurement error effects may induce wrong conclusion. In particular, measurement error effects may affect the control limits and falsely detect out-of-control process parameters, and thus, misleading results might happen. In existing literature, measurement error in continuous random variables was discussed, such as EWMA control charts [10], Shewhart control charts [11], and first-order autoregressive model [12]. However, those approaches simply examined the impact of measurement error but did not correct for measurement error effects in control charts. On the other hand, for *p*-control charts with binary random variables, little methods or discussions for handling measurement error have been available.

The other concern is the implementation of computation for general data analysts. In applications, several packages summarized in Table 1 are commonly used by practitioners. For example, **qcc** and **spc** provide several conventional control charts, such as the Shewhart, EWMA, and CUSUM charts. **MSQC** implements multivariate control charts, including Hotelling *T*^2^, Chi-squared, multivariate EWMA (MEWMA), and multivariate CUSUM (MCUSUM). **qcr** summarizes univariate and multivariate charts and integrates several well-known R packages, such as **qcc** and **MSQC**. Some packages focused on specific scenarios, such as **pbcc** for percentile-based control charts (e.g., [13]). However, those packages were developed by assuming that the data are precisely measured, and they fail to handle error-prone data. In addition, the computational implementation of existing packages requires the normal distribution assumption to obtain the control limit. To fill out the research gap and deal with measurement error effects, we extend the methods proposed by [3, 14] and develop an R package **EATME**, whose main contributions are summarized in Table 1. First, the key advantage of the package **EATME** is that it constructs the corrected EWMA statistic with measurement error effects in continuous or binary random variables corrected, and is useful to derive the reliable control limits, so that in-control (IC) and out-of-control processes can be precisely monitored. In addition, our estimation procedure is based on the Monte Carlo method, which enables us to deal with the distribution-free setting, so that the EWMA statistic is useful to handle binary or continuous random variables without the normal distribution assumption imposed. Furthermore, our package provides various scenarios of control charts, including two-sided control limits or one-sided control limit, which are flexible to achieve users’ requirements. For the computational implementation, the arguments in our functions are similar to those in the existing packages, which enables users to implement the same dataset to different packages and make comparisons easy.

**Table 1:** Comparisons among existing and proposed packages. ‘ME’ represents measurement error correction. ‘DF’ represents the distribution free implementation. ‘STAT’ is the report of some statistics for run length. ‘MC’ is the Monte Carlo implementation. ‘CL’ represents the flexibility of two-sided or one-sided control limits in the control chart.

	Usage				
Packages	ME	DF	STAT	MC	CL
**EATME**	✓	✓	✓	✓	✓
**qcc**	×	×	✓	×	×
**spc**	×	×	✓	×	×
**MSQC**	×	×	✓	×	×
**pbcc**	×	×	✓	×	✓

The remainder is organized as follows. In the Section 2, we introduce the data structure and the basic tool for the construction of control charts. In addition, measurement error models are introduced. In Section 3, we introduce key strategies to correct for measurement error effects and derive corrected control charts. Moreover, we introduce a computational algorithm to evaluate the average run length (ARL). In Section 4, we introduce functions and their arguments in the R package **EATME**. In Section 5, we demonstrate the application of the R package **EATME** and conduct simulation studies to assess the performance of the proposed method. In Section 6, we apply the package to analyze a real dataset. Finally, a general discussion is summarized in Section 7.

## 2 Notation and model

### 2.1 Data structure and EWMA charts

Let *T* denote the monitoring time. For each time *t* = 1, ⋯, *T*, there are *n* subjects. Let *X*_*it*_ for *i* = 1, ⋯, *n* and *t* = 1, ⋯, *T* denote the independent and identically distributed (i.i.d.) random variable. In applications, the random variable *X*_*it*_ can be continuous or binary, where the former case assumes that var(*X*_*it*_) = *σ*^2^, and the latter scenario gives outcome 0 or 1 with *X*_*it*_ = 1 representing a non-conforming product and *X*_*it*_ = 0 standing for a conforming product. The main interest is to monitor the proportion of non-conforming products for binary IC process and monitor variance for continuous IC process.

To accurately monitor IC process and detect OC process parameter, the EWMA control chart is a useful method because it is sensitive in detecting small shifts in process parameters (e.g., [14, 15]). We first explore the scenario that *X*_*it*_ is binary. Let $p_{0}≜P\left(X_{it}=1\right)$ be the parameter of proportion of non-conforming products for IC process, and let $q_{0}≜1-p_{0}=P\left(X_{it}=0\right)$ denote the probability of conforming products for IC process. Given *n* subjects for *T* monitoring times in the IC data, we define the sample proportion ${\hat{p}}_{0,t}≜\frac{1}{n}\sum_{i=1}^{n}X_{it}$ for *t* = 1, ⋯, *T*. Based on the binary random variable and the sample proportion ${\hat{p}}_{0,t}$, the in-control EWMA statistic is given by

$$\begin{array}{c} {\text{EWMA}}_{0,t}=\lambda {\hat{p}}_{0,t}+\left(1-\lambda \right){\text{EWMA}}_{0,t-1} \end{array}$$ (1)

for *t* = 1, ⋯, *T*, where EWMA_0,0_ = *p*_0_ with *p*_0_ being the in-control proportion, and λ ∈ (0, 1] is a pre-specified smoothing parameter.

On the other hand, when *X*_*it*_ is continuous, we first follow the strategy in [3] to use the sign method to convert the continuous variable to the binary variable. After that, we adopt (1) to develop the corresponding EWMA statistic and control limit. Specifically, for a fixed *t* and *j*′ = 1, 2, ⋯, 0.5*n*, define

$$\begin{array}{c} Y_{j^{\prime }t}=\frac{{\left(X_{(2j^{\prime })t}-X_{(2j^{\prime }-1)t}\right)}^{2}}{2}, \end{array}$$ (2)

which satisfies *E*(*Y*_*j*′*t*_) = *σ*^2^. Noting that if *n* is odd, then the last observation will be automatically removed when computing (2) (e.g., [3]). Based on (2), we further define a binary indicator

$$\begin{array}{c} I_{j^{\prime }t}=I\left(Y_{j^{\prime }t}>\sigma^{2}\right) \end{array}$$ (3)

for a fixed *t* and *j*′ = 1, 2, ⋯, 0.5*n*, where I(·) is an indicator function. We now define the *V* statistic, denoted as *V*_*t*_, as the sum of (3) at the *t*th sampling period

$$\begin{array}{c} V_{t}=\sum_{j^{\prime }=1}^{0.5n}I_{j^{\prime }t} \end{array}$$ (4)

for *t* = 1, ⋯, *T*, which follows a binomial distribution Bin(0.5*n*, *p*_0_) with $p_{0}≜P\left(Y_{j^{\prime }t}>\sigma^{2}\right)$. Consequently, the proportion is given by ${\hat{p}}_{0,t}=\frac{1}{0.5n}V_{n}$, and (1) can be taken as a charting statistic.

Finally, based on the EWMA statistic, we develop the control region to monitor the IC process and detect OC data. In our study, we provide one-sided and two-sided control charts, which are respectively given by

$$\begin{array}{c} \left\{ \begin{array}{c} \text{UCL}=p_{0}+L_{1}\sqrt{\frac{p_{0}\left(1-p_{0}\right)\lambda \left\{1-{\left(1-\lambda \right)}^{2t}\right\}}{n(2-\lambda )}}\\ \text{LCL}=0, \end{array}\right. \end{array}$$ (5)

$$\begin{array}{c} \left\{ \begin{array}{c} \text{UCL}=\infty \\ \text{LCL}=p_{0}-L_{2}\sqrt{\frac{p_{0}\left(1-p_{0}\right)\lambda \left\{1-{\left(1-\lambda \right)}^{2t}\right\}}{n(2-\lambda )}}, \end{array}\right. \end{array}$$ (6)

and

$$\begin{array}{c} \left\{ \begin{array}{c} \text{UCL}=p_{0}+L_{3}\sqrt{\frac{p_{0}\left(1-p_{0}\right)\lambda \left\{1-{\left(1-\lambda \right)}^{2t}\right\}}{n(2-\lambda )}}\\ \text{LCL}=p_{0}-L_{4}\sqrt{\frac{p_{0}\left(1-p_{0}\right)\lambda \left\{1-{\left(1-\lambda \right)}^{2t}\right\}}{n(2-\lambda )}}, \end{array}\right. \end{array}$$ (7)

where *L*_1_, *L*_2_, *L*_3_, and *L*_4_ are unknown coefficients. The first one-sided control chart (5) reflects that the the upper control limit (UCL) is provided, the second one-sided control chart (6) considers the finite lower control limit (LCL), and the two-sided control chart (7) gives finite UCL and LCL. Various control charts can be used to address different purposes. For example, when the monitoring time goes by, the OC non-conforming proportion is non-decreasing and is greater than IC non-conforming proportion. Thus, in applications, it is reasonable to set one-sided charts.

### 2.2 Measurement error models

In applications, the random variable *X*_*it*_, either continuous or binary, might be subject to measurement error. That is, the random variable *X*_*it*_ is unobservable, and one can only collect its observed version, denoted $X_{it}^{*}$.

There are two measurement error models to characterize *X*_*it*_ and $X_{it}^{*}$. When *X*_*it*_ and $X_{it}^{*}$ are binary, we first define $p_{0}^{*}≜P\left(X_{it}^{*}=1\right)$ and $q_{0}^{*}≜1-p_{0}^{*}=P\left(X_{it}^{*}=0\right)$ as the in-control observed version of *p*_0_ and *q*_0_, respectively. Then by the technique of law of total probability, the relationship of $p_{0}^{*}$ and *p*_0_ is given by

$$\begin{array}{c} p_{0}^{✱}=\pi_{11}p_{0}+\pi_{10}q_{0}. \end{array}$$ (8)

Similarly, $q_{0}^{*}$ and *q*_0_ can be characterized as

$$\begin{array}{c} q_{0}^{✱}=\pi_{01}p_{0}+\pi_{00}q_{0}. \end{array}$$ (9)

The matrix form of (8) and (9) is given by

$$\begin{array}{c} (\begin{array}{c} p_{0}^{✱}\\ q_{0}^{✱} \end{array})=\Pi (\begin{array}{c} p_{0}\\ q_{0} \end{array}), \end{array}$$ (10)

where $\Pi =(\begin{array}{cc} \pi_{11} & \pi_{10}\\ \pi_{01} & \pi_{00} \end{array})$ is the 2 × 2 (mis)classification matrix with probability $\pi_{kj}=P\left(X_{it}^{*}=k|X_{it}=j\right)$, and the sum of elements in each column is equal to one, i.e., *π*_11_ + *π*_01_ = 1 and *π*_10_ + *π*_00_ = 1. Following the similar discussion in [16], we assume that **Π** has the spectral decomposition **Π** = **ΩDΩ**^−1^, where **D** is the diagonal matrix with diagonal elements being the eigenvalues of **Π**, and *Ω* is the corresponding matrix of eigenvectors.

If *X*_*it*_ and $X_{it}^{*}$ are continuous, then we employ the classical measurement error model

$$\begin{array}{c} X_{it}^{✱}=X_{it}+\epsilon_{it}, \end{array}$$ (11)

where *ϵ*_*it*_ is the noise term satisfying *E*(*ϵ*_*it*_) = 0 and $\text{var}\left(\epsilon_{it}\right)=\sigma_{\epsilon }^{2}$ that can be reparameterized as *δσ*^2^ for some positive constant *δ*. In addition, the independence of *ϵ*_*it*_ and *X*_*it*_ is also assumed. Following the discussion in Section 2.1, the continuous error-prone random variable $X_{it}^{*}$ can be transformed to the binary one. Specifically, similar to (2) and (3), we have

$$\begin{array}{c} V_{t}^{✱}=\sum_{j^{\prime }=1}^{0.5n}I_{j^{\prime }t}^{✱} \end{array}$$

that follows a binomial distribution $\text{Bin}(0.5n,p_{0}^{*})$ with $p_{0}^{*}≜P\left(Y_{j^{\prime }t}^{*}>\left(1+\delta \right)\sigma^{2}\right)$, where $I_{j^{\prime }t}^{*}=I\left(Y_{j^{\prime }t}^{*}>\left(1+\delta \right)\sigma^{2}\right)$ and $Y_{j^{\prime }t}^{*}=\frac{{\left(X_{(2j^{\prime })t}^{*}-X_{(2j^{\prime }-1)t}^{*}\right)}^{2}}{2}$. Based on this transformation, one can apply (10) to connect random variables $V_{t}^{*}$ and *V*_*t*_.

In the following presentation, to focus the discussion on correcting for measurement error effects and constructing the corrected control charts, we take parameters **Π** or $\sigma_{\epsilon }^{2}$ in measurement error models (10) or (11) as known constants. In practice, if they are unknown, then one needs additional information, such as repeated measurements or validation data, to estimate them [16]. Otherwise, one may conduct sensitivity analyses in data analysis, whose idea is to specify various values in **Π** or $\sigma_{\epsilon }^{2}$ and examine the impact of measurement error effects. The detailed demonstration is deferred to Section 6.

## 3 Overview of methodology

### 3.1 Correction of measurement error effects

As commented by [9], ignoring measurement error effects may induce tremendous biases for the estimators and derive misleading conclusion. It is expected that directly using error prone data $X_{it}^{*}$ to the EWMA statistic (1) would incur unconvincing control limits and delay OC detection. As a result, it is crucial to correct for measurement error effects.

According to the discussion in Section 2.2, we know that binary or continuous random variables and their observed versions can be eventually formulated by (10), it suffices to consider the correction based on (10). To see this, following the strategy in [14] and taking the inverse of **Π** on both sides of (10) yield that

$$\begin{array}{c} \Pi^{-1}(\begin{array}{c} p_{0}^{✱}\\ q_{0}^{✱} \end{array})=(\begin{array}{c} p_{0}\\ q_{0} \end{array}). \end{array}$$

It suggests that the proportion *p*_0_ can be expressed as

$$\begin{array}{c} p_{0}=\frac{p_{0}^{✱}-\pi_{10}}{1-\pi_{10}-\pi_{01}}. \end{array}$$ (12)

Consequently, (12) shows that the *corrected* random variable is given by

$$\begin{array}{c} X_{it}^{✱✱}≜\frac{X_{it}^{✱}-\pi_{10}}{1-\pi_{10}-\pi_{01}}, \end{array}$$

and thus, the corrected sample proportion is denoted as ${\hat{p}}_{0,t}^{**}=\frac{1}{n}\sum_{i=1}^{n}X_{it}^{**}$. Therefore, we can replace ${\hat{p}}_{0,t}$ in (1) by ${\hat{p}}_{0,t}^{**}$ to derive the corrected EWMA statistic.

### 3.2 Computational algorithm for coefficients of control limits and run length

Note that *L*_1_ or *L*_2_ in one-sided control charts (5) and (6) are usually unknown, and it is crucial to estimate it by the data-driven approach. To address the estimation of *L*_1_ and *L*_2_, we employ the Monte Carlo method, which is summarized in Algorithm 1. The detailed descriptions can be referred to [14]. From this algorithm, in addition to obtaining the estimated coefficient of control limits, we also evaluate the in-control run length *t*_0,*m*_ and compute the in-control average run length (ARL_0_), the in-control median run length (MRL_0_), and the in-control standard deviation run length (SDRL_0_).

**Algorithm 1:** Monte Carlo implementation used to find coefficients of the control limit and statistics for two one-sided corrected control charts

**Step 1:** Given in-control *p*_0_ and **Π**, $p_{0}^{*}$ is calculated by formula (10);

**Step 2:** Set λ, *n*, and a value of pre-specified in-control average run length ${\text{ARL}}_{0}^{*}$;

**Step 3:** Set *a* < *L*_1_ (or *L*_2_) < *b* with *a* = 0.01 and *b* = 10 (say);

**Step 4:** Monte Carlo procedure:

**for** step (*m* + 1) with *m* = 1, 2, ⋯, *M* and set *M* = 10001 (say) **do**

 **Step 4.1:** Let ${\text{EWMA}}_{0,0}=p_{0}^{**}$ and *t* = 1;

 **Step 4.2:** Based on the observed and misclassified $X_{it}^{*}$, calculate ${\hat{p}}_{0,t}^{**}≜\frac{1}{n}\sum_{i=1}^{n}\frac{X_{it}^{*}-\pi_{10}}{1-\pi_{10}-\pi_{01}}$;

  • If *t* = 1, ${\text{EWMA}}_{0,1}=\lambda {\hat{p}}_{0,1}^{**}+\left(1-\lambda \right){\text{EWMA}}_{0,0}$;

  • If *t* ≠ 1, ${\text{EWMA}}_{0,t}=\lambda {\hat{p}}_{0,t}^{**}+\left(1-\lambda \right){\text{EWMA}}_{0,t-1}$.

 **Step 4.3:** Given *L*_1_ (or *L*_2_) and compute UCL (or LCL);

  • If EWMA_0,*t*_ ≥ UCL (or ≤ LCL), then take $t_{0,m}≜t$ as a run length and *m* ← *m* + 1.

    Go to Step 4.1;

  • If EWMA_0,*t*_ ≤ UCL (or ≥LCL), then *t* ← *t* + 1. Go to Step 4.2.

**end**

**Step 5:**

Calculate $\frac{1}{M}\sum_{m=1}^{M}t_{0,m}$ and take it as the estimate of the nominal ARL_0_, which is denoted as ${\hat{\text{ARL}}}_{0}$. Finally, determine *L*_1_ (or *L*_2_) by minimizing $|{\text{ARL}}_{0}^{*}-{\hat{\text{ARL}}}_{0}|<1$ subject to *a* < *L*_1_ (or *L*_2_)<*b*. Moreover, with a given sequence of in-control run lengths *t*_0,*m*_ with *m* = 1, ⋯, *M*, we compute the median run length ${\hat{\text{MRL}}}_{0}=\text{median}\left\{t_{0,m}:m=1,\ldots ,M\right\}$ and the standard deviation run length ${\hat{\text{SDRL}}}_{0}=\frac{1}{M-1}\sum_{m=1}^{M}{\left(t_{0,m}-{\hat{\text{ARL}}}_{0}\right)}^{2}$.

On the other hand, under two-sided control charts (7), there are two unknown coefficients *L*_3_ and *L*_4_. To simultaneously estimate *L*_3_ and *L*_4_, we adopt Algorithm 1 with some modifications. Specifically, we first treat LCL in (7) to be 0, and apply Algorithm 2 to find *L*_3_ for UCL. Unlike Step 5 in Algorithm 1, here the estimated value of *L*_3_ and the resulting estimated value of ${\hat{\text{ARL}}}_{0}$ should satisfy $|2{\text{ARL}}_{0}^{*}-{\hat{\text{ARL}}}_{0}|<1$ under a pre-specified in-control ARL (${\text{ARL}}_{0}^{*}$). Once estimated value of *L*_3_ is obtained, we repeat the same procedure in Algorithm 1 to estimate *L*_4_, with $|{\text{ARL}}_{0}^{*}-{\hat{\text{ARL}}}_{0}|<1$ satisfied. To show the clear procedure, we summarize the pseudo-code in Algorithm 2.

**Algorithm 2:** Monte Carlo implementation used to find coefficients of the control limit and statistics for two-sided corrected control charts

**Stage I: Estimation of *L*_1_**;

**Step 1:** Given in-control *p*_0_ and **Π**, $p_{0}^{*}$ is calculated by formula (10);

**Step 2:** Set λ, *n*, and a value of ARL_0_;

**Step 3:** Set *a* < *L*_1_ < *b* with *a* = 0.01 and *b* = 10 (say);

**Step 4:** Monte Carlo procedure:

**for** step (*m* + 1) with *m* = 1, 2, ⋯, *M* and set *M* = 10001 (say) **do**;

 **Step 4.1:** Let ${\text{EWMA}}_{0,0}=p_{0}^{**}$ and *t* = 1;

 **Step 4.2:** Based on the observed and misclassified $X_{it}^{*}$, calculate ${\hat{p}}_{0,t}^{**}≜\frac{1}{n}\sum_{i=1}^{n}\frac{X_{it}^{*}-\pi_{10}}{1-\pi_{10}-\pi_{01}}$;

  • If *t* = 1, ${\text{EWMA}}_{0,1}=\lambda {\hat{p}}_{0,1}^{**}+\left(1-\lambda \right){\text{EWMA}}_{0,0}$;

  • If *t* ≠ 1, ${\text{EWMA}}_{0,t}=\lambda {\hat{p}}_{0,t}^{**}+\left(1-\lambda \right){\text{EWMA}}_{0,t-1}$.

 Step 4.3: Given *L*_1_ and compute UCL;

  • If EWMA_0,*t*_ ≥ UCL, then take $t_{0,m}≜t$ as a run length and *m* ← *m* + 1.

   Go to Step 4.1;

  • If EWMA_0,*t*_ ≤ UCL, then *t* ← *t* + 1. Go to Step 4.2.

**end**

**Step 5:** Calculate $\frac{1}{M}\sum_{m=1}^{M}t_{0,m}$ and take it as the estimate of the nominal ARL_0_, which is denoted as ${\hat{\text{ARL}}}_{0}$. Finally, determine *L*_1_ by minimizing $|2{\text{ARL}}_{0}-{\hat{\text{ARL}}}_{0}|<1$ subject to *a* < *L*_1_ < *b*.

**Stage II: Estimation of *L*_2_**;

**Step 6:** Set *a* < *L*_2_ < *b*, with *a* = 0.01 and *b* = 5;

**Step 7:** Monte Carlo procedure:

**for** step (*m* + 1) with *m* = 1, 2, ⋯, *M* and set *M* = 10001 (say) **do**

 **Step 7.1:** Let ${\text{EWMA}}_{0,0}=p_{0}^{**}$ and *t* = 1;

 **Step 7.2:** Based on the observed and misclassified $X_{it}^{*}$, calculate ${\hat{p}}_{0,t}^{**}≜\frac{1}{n}\sum_{i=1}^{n}\frac{X_{it}^{*}-\pi_{10}}{1-\pi_{10}-\pi_{01}}$;

  • If *t* = 1, ${\text{EWMA}}_{0,1}=\lambda {\hat{p}}_{0,1}^{**}+\left(1-\lambda \right){\text{EWMA}}_{0,0}$;

  • If *t* ≠ 1, ${\text{EWMA}}_{0,t}=\lambda {\hat{p}}_{0,t}^{**}+\left(1-\lambda \right){\text{EWMA}}_{0,t-1}$.

 **Step 7.3:** Given estimated *L*_1_ from Step 5 and *L*_2_ in Step 6, compute UCL and LCL;

  • If EWMA_0,*t*_ ≥ UCL or EWMA_0,*t*_ ≤ LCL, then take $t_{0,m}≜t$ as a run length and *m* ← *m* + 1.

   Go to Step 7.1;

  •If LCL ≤ EWMA_0,*t*_ ≤ UCL, then *t* ← *t* + 1. Go to Step 7.2.

**end**

**Step 8:** Calculate $\frac{1}{M}\sum_{m=1}^{M}t_{m}$, take it to be estimator of the ${\text{ARL}}_{0}$, and is denoted as ${\hat{\text{ARL}}}_{0}$. And determine *L*_2_ by minimizing $|{\text{ARL}}_{0}-{\hat{\text{ARL}}}_{0}|<1$, subject to *a* < *L*_2_ < *b*.

## 4 Description of EATME

In this section, we introduce several main functions in the package **EATME** for the implementation of the estimation method in Section 3, which can be classified to several categories in Table 2. In addition, the arguments of the functions are summarized in Table 3. To the end, we briefly describe them in the following subsections.

**Table 2:** Summary of the functions in the package EATME.

Categories	Functions	Purpose
Synthetic data	ME_data_generate	generate the synthetic data by the simulation.
Data transformation	cont_to_disc_V	implement (4) to transform the continuous variables.
Statistics	EWMA_p_one_LCL	implement Algorithms 2 and 2 to (1) and derive the EWMA statistic as well as control limits (5), (6), or (7).
	EWMA_p_one_UCL	
	EWMA_p_two	
Charts	EWMA_p_chart_one_LCL	implement the variables to obtain the resulting control charts for IC and OC data.
	EWMA_p_chart_one_UCL	
	EWMA_p_chart_two	

**Table 3:** Summary of the arguments of the functions in the package EATME.

Arguments	Explanations
ICdata	The in-control data for attributes.
OCdata	The out-of-control data for attributes.
n	A sample size *n* in each time period.
m	A number of sample periods *T* in the data.
p	A in-control probability *p*_0_ of the unobserved defectives.
lambda	An EWMA smooth constant λ in (1), which is a scalar in (0, 1].
pi1	The proportion *π*_11_ in (9).
pi2	The proportion *π*_00_ in (9).
ARL0	A pre-specified ARL of a control chart in the in-control process.
M	The number of the Monte Carlo repetitions in Algorithms 2 and 2.
past	The proportion *p*_0_ or $p_{0}^{*}$ in the IC process.
var.p	Variance of the continuous random variables *σ*^2^ in the IC data.
error	The tolerance for the absolute difference between an iterated ARL
	value and pre-specified ARL0.

### 4.1 Category 1: Simulated data

To make users understand the data structure of SPC with measurement error accommodated, we introduce the function ME_data_generate, which randomly generates the synthetic binary variables. The implementation is given by

ME_data_generate(p,n,m,pi1,pi2=pi1).

The basic idea of this function is to apply (10) to generate $X_{it}^{*}$, provided that the true proportion *p*_0_ is given. Consequently, the outputs of this function include

- real_data: The generated data *X*_*it*_ without measurement error.
- obs_data: The generated data $X_{it}^{*}$ with measurement error.

### 4.2 Category 2: Data transformation

Since our main method is based on the proportion-based EWMA method (1), to apply our method to deal with the continuous random variables, we require to do the transformation (4) and convert the continuous variables to the discrete ones. As a result, the function cont_to_disc_V is used to achieve this goal. The implementation is given by

cont_to_disc_V(ICdata,OCdata,var.p=NULL).

The function produces (4) based on ICdata and OCdata, where the transformed variables under IC and OC are recorded by V0 and V1, respectively; the process proportion *P*(*Y*_*j*′*t*_ > *σ*^2^) under IC and OC data recorded by p0 and p1, respectively.

### 4.3 Category 3: Statistics

Given the random variables that are either precisely measured or error contaminated, we aim to apply the EWMA statistic (1) to develop the control chart and detect OC data by implementing the following functions:

EWMA_p_one_UCL(past, lambda, n, pi1 = 1, pi2 = pi1, ARL0, M = 500, error = 10),

EWMA_p_one_LCL(past, lambda, n, pi1 = 1, pi2 = pi1, ARL0, M = 500, error = 10),

and

EWMA_p_two(past, lambda, n, pi1 = 1, pi2 = pi1, ARL0, M = 500, error = 10).

Basically, those three functions require to input proportion of precisely measured defectives *p*_0_ or error-prone defectives $p_{0}^{*}$ in the argument past. The arguments pi1 and pi2 are used to reflect the correctness of measurement error, as shown in (12). In particular, pi1 = 1 and pi2 = 1 give no correction. In addition, those three functions are used to implement the Monte Carlo computation with repetition M in Algorithms 1 and 2 and provide estimated IC ARL (hat_ARL0), IC median run length (hat_MRL0), and IC standard deviation of run length (hat_SDRL0). The main difference among those three functions is that the first two functions reflect one-sided control charts (5) or (6) with the resulting coefficients *L*_1_ (L1) or *L*_2_ (L2), UCL (UCL), and LCL (LCL) reported, respectively. In contrast, the last function gives the information of the two-sided control chart (7) and reports the resulting coefficients *L*_3_ (recorded as L1) and *L*_4_ (recorded as L2) as well as UCL and LCL.

### 4.4 Category 4: Charts

Finally, to assess the performance of IC monitoring and OC detection, we apply the following functions to display the IC and OC control charts:

EWMA_p_chart_one_UCL(ICdata, OCdata, lambda, n, pi1 = 1, pi2 = pi1, ARL0, M = 500, error = 10),

EWMA_p_chart_one_LCL(ICdata, OCdata, lambda, n, pi1 = 1, pi2 = pi1, ARL0, M = 500, error = 10),

and

EWMA_p_chart_two(ICdata, OCdata, lambda, n, pi1 = 1, pi2 = pi1, ARL0, M = 500, error = 10).

Similar to the functions in Section 4.3, the IC data (ICdata) and OC data (OCdata) can be precisely measured or error-contaminated, and two arguments pi1 and pi2 reflect the correctness of measurement error in (12). All functions show IC and OC control charts, where horizontal solid lines represent upper control limit (UCL) and lower control limit (LCL) with the corresponding values labelled, black solid dots are detection of IC data, and red solid dots are detection of OC data. The first two functions give one-sided control charts based on (5) or (6), respectively; the last function produces the two-sided control chart from (7).

## 5 Simulation and demonstration of programming code

In this section, we demonstrate functions in the package **EATME** to conduct simulation studies for binary and continuous random variables, respectively.

### 5.1 Demonstration of binary random variables

In this subsection, we demonstrate simulation studies for binary random variables. We first adopt the function ME_data_generate to generate error-prone data. Specifically, we consider *T* = 50 sample periods and each time contains *n* = 20 samples. The in-control proportion *p*_0_ is specified as 0.4 for the generation of *X*_*jt*_. Next, we generate the error-prone data $X_{jt}^{*}$ by (10) with *π*_11_ = *π*_00_ = 0.9. To generate out-of-control process, we consider 20 time periods, and specify *p*_1_ = 0.6 to generate the unobserved random variable. Similar to the in-control process, *π*_11_ = *π*_00_ = 0.9 is given in (10) to generate out-of-control error-prone samples.

The detailed implementation code for data generation is given below, where discrete_IC and discrete_OC are in-control and out-of-control samples, respectively, $real_data is *X*_*jt*_ and $obs_data reflects $X_{jt}^{*}$.


> (discrete_IC = ME_data_generate(0.4,20,50,0.9,0.9))
$real_data
 [1]  7  8  8  8  7  8  7  8  6  7  5 12 10  6 11 10  4  6 10  9 10
9  6  6  8
[26]  6  8  5  9 10  8  9  7 13  8 10 10  7  8  7 11 12  6  9  8 11
5  4  8 11

$obs_data
 [1]  6 11 10  8  7  7  8  9  4  5  5 10 10  7 10 10  8  7  9  9  8 
 11  8  7 10
[26]  8  4  6  9  9  8  9  7 13 10  8  9  9  8 10 11 14  9 11  9  9
4  5 10 11

$n
[1] 20

> (discrete_OC = ME_data_generate(0.6,20,20,0.9,0.9))
$real_data
 [1] 14  9 13 11 13 11 11 13 12 13  8 14 13 10  9 12  9 15  5 10

$obs_data
 [1] 14  9 12 10 15 10 12  9 12 13  8 13 12 11 10 13  8 15  4 10

$n
[1] 20


After that, we now aim to construct the one-sided EWMA-p chart for monitoring process defective and examine the impact of measurement error. Based on the synthetic data, we use the mean of defective proportion to estimate *p*_0_, and use the function EWMA_p_one_UCL to find the control limits. The implementation code is given below, where CL provides the result based on the true random variable *X*_*jt*_, CL_s reflects the results based on error-prone data $X_{jt}^{*}$ without measurement error correction, and CL_ss gives numerical results with measurement error correction.


> CL = EWMA_p_one_UCL(mean(discrete_IC$real_data/discrete_IC$n),
+          0.05, discrete_IC$n,1,1,370.4,10000,1)
> CL_s = EWMA_p_one_UCL(mean(discrete_IC$obs_data/discrete_IC$n),
+          0.05, discrete_IC$n,1,1,370.4,10000,1)
> CL_ss = EWMA_p_one_UCL(mean(discrete_IC$obs_data/discrete_IC$n),
+         0.05, discrete_IC$n,0.9,0.9,370.4,10000,1)
> cbind(CL,CL_s,CL_ss)
         CL        CL_s      CL_ss    
L1       2.194824  2.188781  1.742764 
hat_ARL0 371.0827  369.5501  369.7929 
hat_MRL  247       248       245.5    
hat_SDRL 397.1297  391.405   391.5981 
UCL      0.4344343 0.4546285 0.4334463


According to the numerical results summarized above, we observe that the error-corrected UCL CL_ss$UCL is more reliable than the UCL without measurement error correction CL_s$UCL because of closeness with the value CL$UCL based on true *X*_*jt*_.

**Fig 1:**
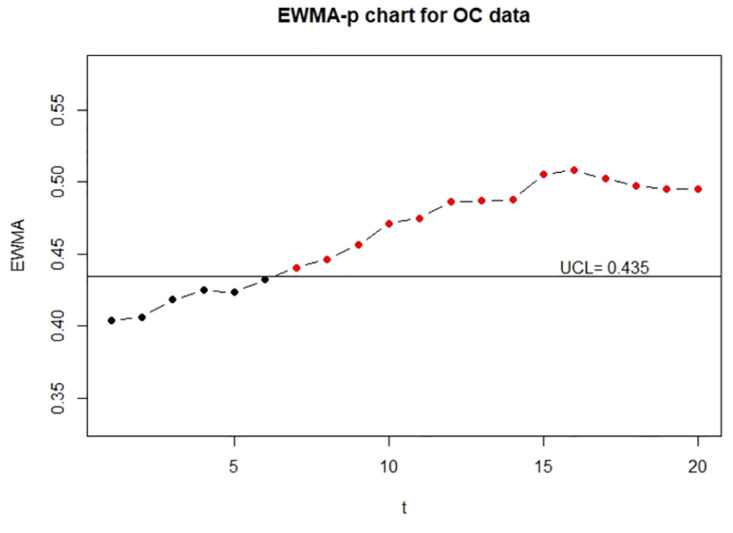
The control charts for the error-free discrete data. The left panel is for the IC data and the right panel is for the OC data.

**Fig 2:**
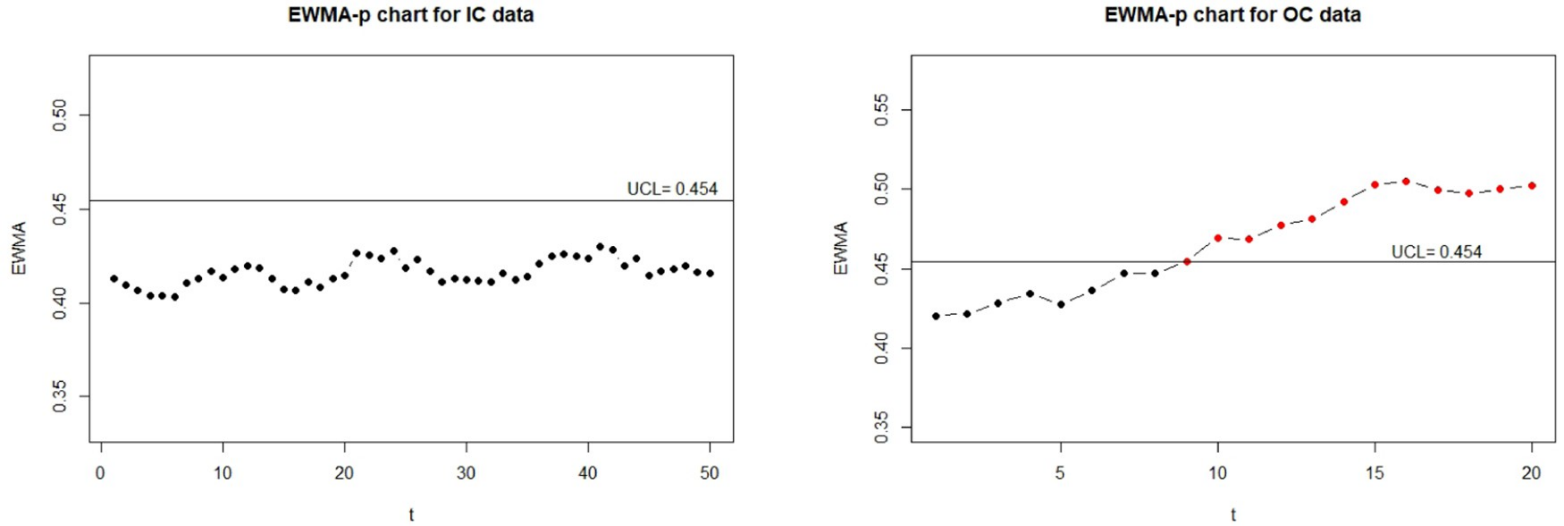
The control charts for the error-prone discrete data. The left panel is for the IC data and the right panel is for the OC data.

**Fig 3:**
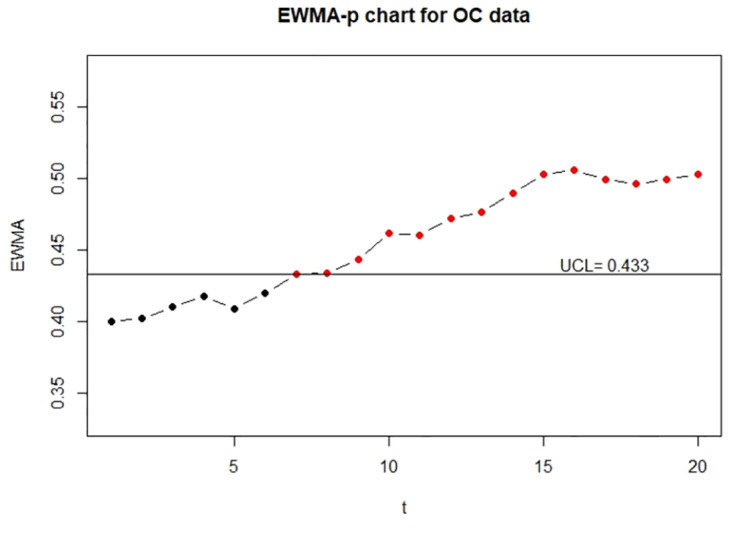
The control charts for the error-corrected discrete data. The left panel is for the IC data and the right panel is for the OC data.


Moreover, for the visualization, we adopt the function EWMA_p_chart_one_UCL to show the one-sided control charts with finite UCL under the true data, error-prone data without correction, and error-prone data with correction. The implementations are described below and the resulting charts are displayed in Figs 1–3.

> EWMA_p_chart_one_UCL(discrete_IC$real_data,discrete_OC$real_data,
+          0.05, discrete_IC$n,1,1,370.4,10000,1)

> EWMA_p_chart_one_UCL(discrete_IC$obs_data,discrete_OC$obs_data,
+          0.05, discrete_IC$n,1,1,370.4,10000,1)

> EWMA_p_chart_one_UCL(discrete_IC$obs_data,discrete_OC$obs_data,
+          0.05, discrete_IC$n,0.9,0.9,370.4,10000,1)


From Figs 1 and 3, we can see that two control charts detect the seventh out-of-control sample is outside of the upper control limits. It shows that the corrected control chart is valid to deal with measurement error and recovers the detective results to the error-free ones. Hence, we conclude that the corrected control chart is a valid method to monitor the process defective. On the other hand, from Fig 2, we can see that the control chart without measurement error correction defers the OC detection to the ninth sample, which might be an evidence that the control chart without measurement error correction falsely detect out-of-control parameters. It also shows that measurement error effects may affect the estimation of UCL.

**Fig 4:**
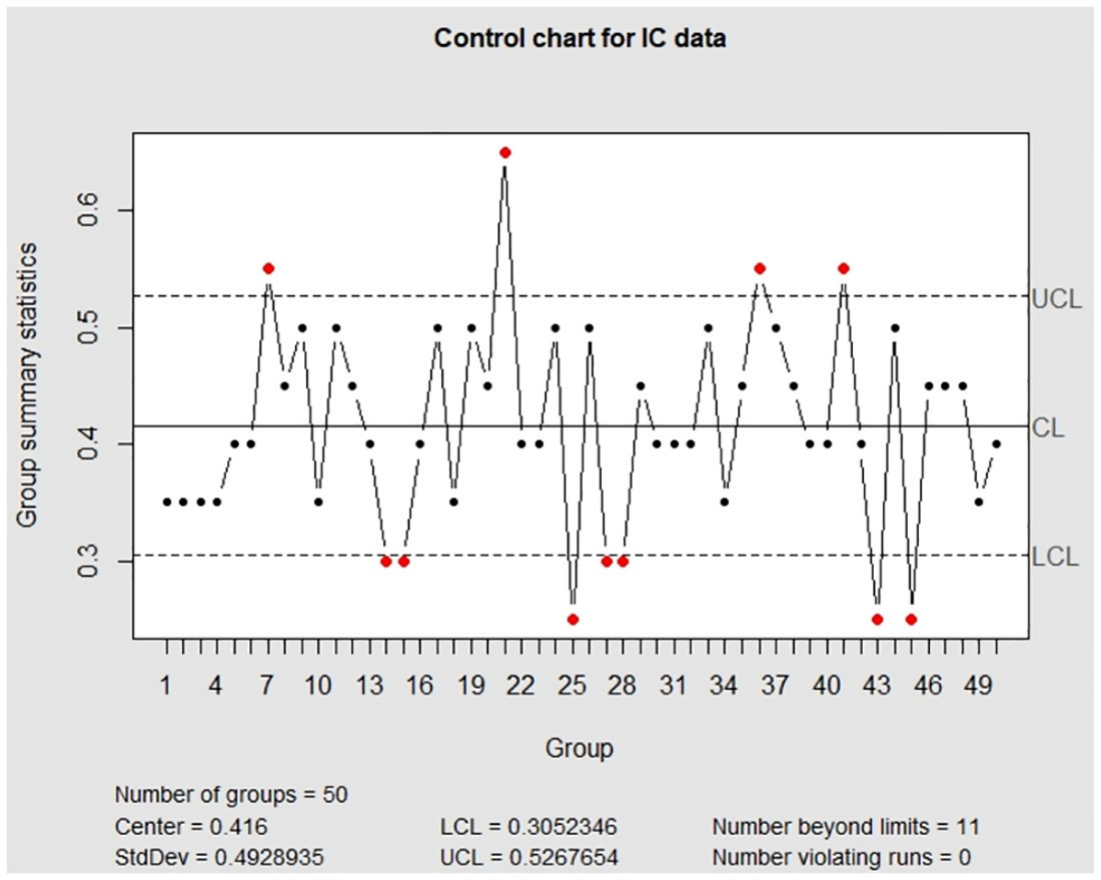
The control charts obtained by the package qcc for the error-prone discrete data. The left panel is for the IC data and the right panel is for the OC data.

Moreover, to show the advantage of the package **EATME**, we primarily examine the package **qcc** in Table 1 and compare its performance with our package because the implementation of the package **qcc** is similar to others. Under the same scenario that the variables are subject to measurement error, we observe from Fig 4 that the IC and OC control charts look fluctuating and are different from the relatively flat trends in Fig 2. In addition, there are 11 false alarm points in the IC control chart and 14 OC detection. According to Table 4, the UCL derived by the package **qcc** is larger than UCL from the package **EATME**, which reflects that the package **qcc** induces wider monitoring region.

**Table 4:** Simulation results and comparisons for the binary data in Section 5.1. ‘○’ reflects the implementation of truly unobserved data. ‘×’ indicates the implementation of error-prone data without measurement error correction. ‘−’ represents no value. A pair (*x*, *y*) in the column (*π*_11_, *π*_00_) is the implementation of the corrected control chart with parameter values (*π*_11_, *π*_00_) = (*x*, *y*) in (10) accommodated.

Packages	(*π*_11_, *π*_00_)	Output values					
		L1	ARL0	MRL	SDRL	LCL	UCL
**EATME**	°	2.194	371.083	247	397.130	−	0.434
	×	2.189	369.550	248	391.405	−	0.455
	(0.907, 0.924)	1.742	369.793	245.5	391.598	−	0.433
**qcc**	×	−	−	−	−	0.305	0.527

### 5.2 Demonstration of continuous random variables

In this subsection, we focus the discussion on continuous random variables and the construction of corrected control charts.

We start by generating in-control continuous random variables from the exponential distribution with mean 20 for 100 sample periods and 15 samples in each time, and generate out-of-control samples from exponential distribution with mean 30 and time period 20. To generate the error-prone data, we adopt the measurement error model (11) with the noise term *ϵ*_*it*_ generated from a normal distribution *N*(0, 30). We summarize the generation in the following code, where conti_IC and conti_OC are in-control and out-of-control true continuous random variables, respectively, and ME_conti_IC and ME_conti_OC are error-prone in-control and out-of-control data separately.


> conti_IC = matrix(rexp(1500,1/20),ncol = 15)
> conti_OC = matrix(rexp(300,1/30),ncol = 15)
> ME_conti_IC = conti_IC+matrix(rnorm(1500,0,sqrt(30)),ncol = 15)
> ME_conti_OC = conti_OC+matrix(rnorm(300,0,sqrt(30)),ncol = 15)


To implement the proposed method, we need to adopt cont_to_disc_V to transform continuous random variables to discrete ones, as shown in (2). The following code and output show the transformation for true random variable and error-prone random variable, labeled by M and M_ME, respectively.


> (M = cont_to_disc_V(conti_IC,conti_OC))
$V0
  [1] 3 1 1 1 4 4 2 2 0 0 3 2 3 2 3 4 1 2 2 1 2 1
      1 1 3 2 1 1 1 2 2 2 1 1 0 3 0 1 1 3 3 2 1 1
 [45] 0 0 3 1 0 1 2 1 0 3 0 1 2 2 1 1 3 3 3 2 2 3 
      1 2 1 1 4 4 0 2 1 2 1 1 1 1 1 0 1 2 1 1 2 1
 [89] 4 1 4 3 2 3 4 4 1 2 3 0

$V1
 [1] 3 2 1 6 2 1 3 5 2 3 3 2 2 1 2 2 3 3 1 1

$p0
[1] 0.2471429

$p1
[1] 0.3428571

$n
[1] 7

> (M_ME = cont_to_disc_V(ME_conti_IC,ME_conti_OC))
$V0
  [1] 3 2 1 1 3 3 2 2 0 1 3 3 2 2 3 4 2 1 2 2 2 1 
      2 1 3 4 1 1 0 2 2 3 1 2 0 3 1 1 2 2 4 2 1 1
 [45] 2 0 3 1 2 1 2 1 2 3 0 1 3 1 1 1 3 2 5 1 2 3 
      2 3 2 1 3 2 1 2 2 2 2 1 0 2 1 2 3 3 2 1 3 0
 [89] 4 1 4 4 2 2 3 3 2 3 2 0

$V1
 [1] 3 3 2 6 1 1 3 5 1 3 4 1 3 1 1 3 4 3 2 2

$p0
[1] 0.2771429

$p1
[1] 0.3714286

$n
[1] 7


Given the true and error-prone data, we now construct the control chart. Moreover, for the measurement error correction, we specify *π*_11_ = 0.907 and *π*_00_ = 0.924. To demonstrate two-sided control chart in in this section, we can use the function EWMA_p_two with arguments lambda and ARL_0 specified as 0.05 and 370.4, respectively, to find the control limits and compute run length. The implementation of EWMA_p_two with/without measurement error correction is described below, where CL, CL_s, and CL_ss reflect the same definitions as those in Section 5.1:


> CL = EWMA_p_two(M$p0,0.05,M$n,1,1,370.4,1000,10)
> CL_s = EWMA_p_two(M_ME$p0,0.05,M$n,1,1,370.4,1000,10)
> CL_ss = EWMA_p_two(M_ME$p0,0.05,M$n,0.907,0.924,370.4,1000,10)
> 
> cbind(CL,CL_s,CL_ss)
         CL        CL_s      CL_ss    
L1       2.577901  2.563372  2.018405 
L2       2.441503  2.472614  1.994251 
hat_ARL0 375.107   361.412   379.456  
hat_MRL  256       240       257      
hat_SDRL 378.6735  377.4985  392.9455 
UCL      0.3144429 0.3464827 0.3058459
LCL      0.1834037 0.2101616 0.1770399


Similar to the discussion in Section 5.1, the error-corrected control limits CL_ss$UCL and CL_ss$LCL are close to values CL$UCL and CL$LCL based on true variable *X*_*jt*_, yielding reliable result and showing the suitable correction of measurement error effects.

**Fig 5:**
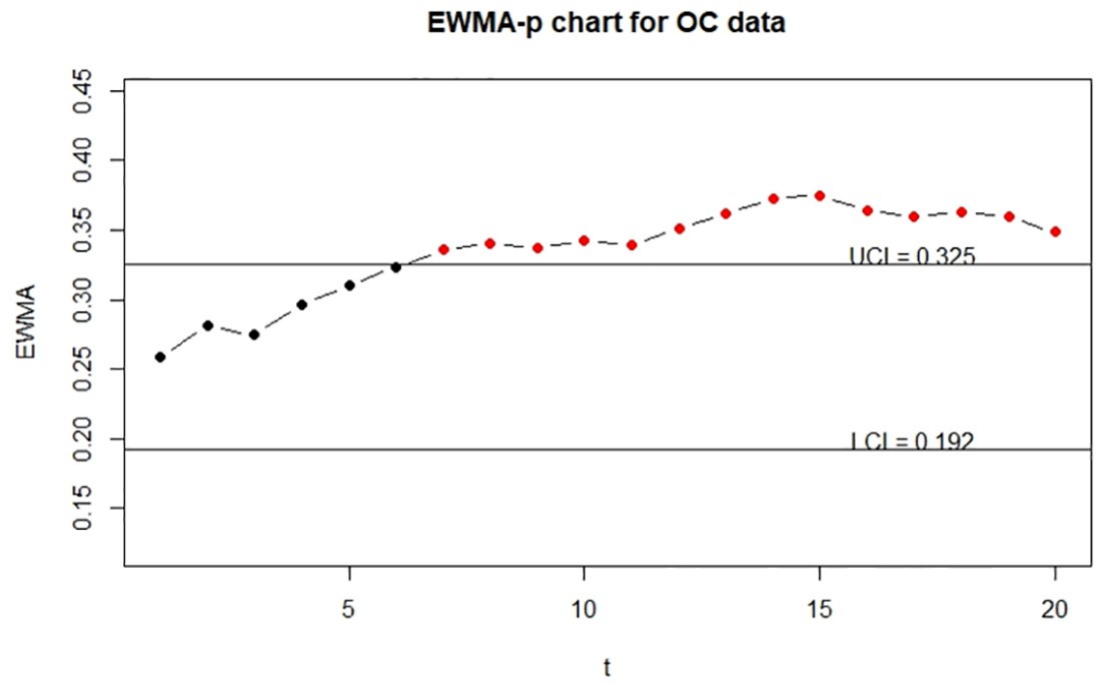
The control charts for the error-free continuous data. The left panel is for the IC data and the right panel is for the OC data.

**Fig 6:**
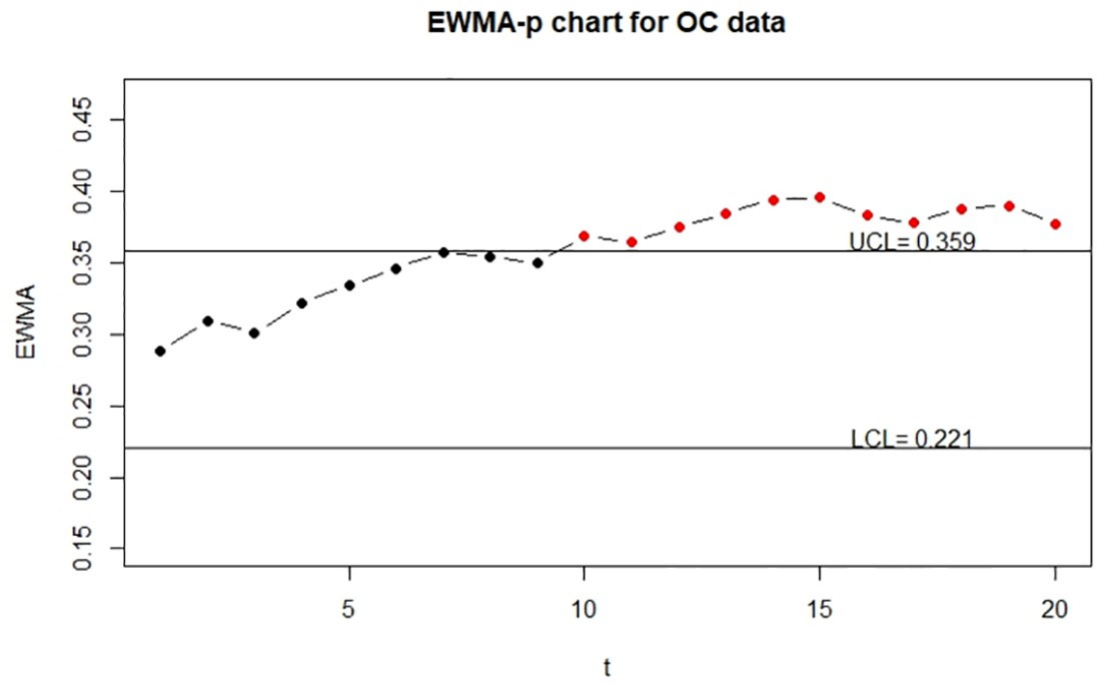
The control charts for the error-prone continuous data. The left panel is for the IC data and the right panel is for the OC data.

**Fig 7:**
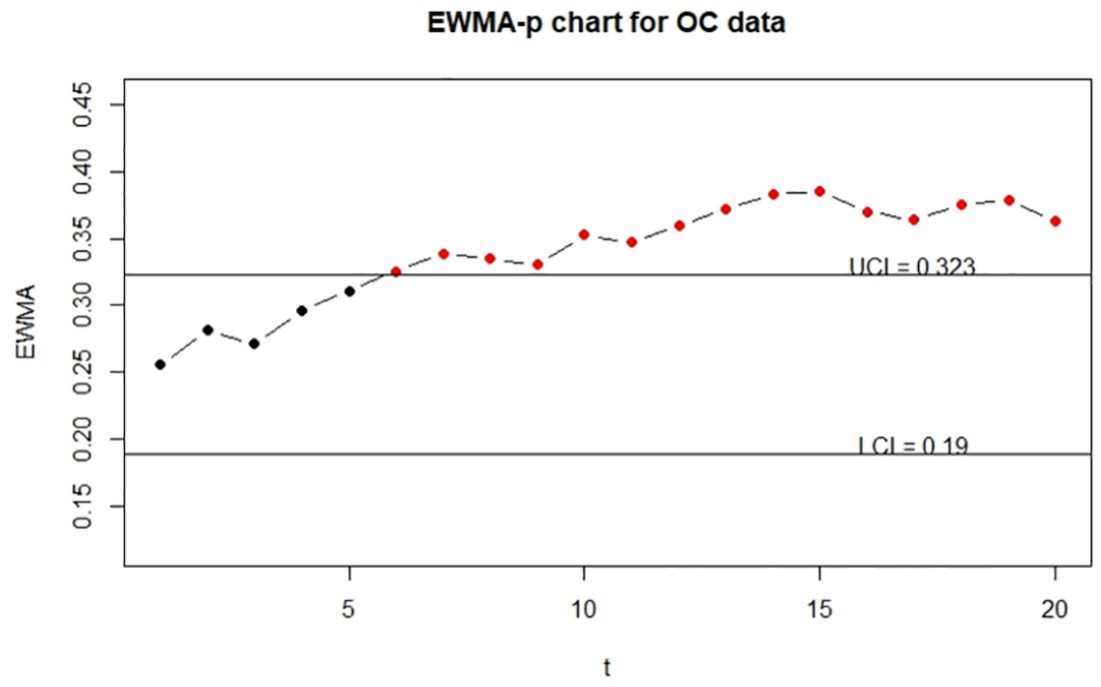
The control charts for the error-corrected continuous data. left panel is for the IC data and the right panel is for the OC data.


To show the visualization of control charts, we demonstrate the function EWMA_p_chart_two to display the two-sided EWMA-p charts for the IC and OC data. The resulting charts are displayed in Figs 5–7.

> EWMA_p_chart_two(M$V0,M$V1,0.05,M$n,1,1,370.4,500,10)

> EWMA_p_chart_two(M_ME$V0,M_ME$V1,0.05,M$n,1,1,370.4,500,10)

> EWMA_p_chart_two(M_ME$V0,M_ME$V1,0.05,M$n,0.907,0.924,370.4,500,10)


From Figs 5 and 7, we can see that control charts signal a shift at the seventh and sixth sample for out-of-control data, respectively, it indicates that the corrected control chart shows the same detection as the control chart obtained by the true random variable. On the contrary, we can see from Fig 6 that the control chart without measurement error correction signals a shift at the tenth sample for out-of-control data, which is an evidence that the control chart without correction may produce false detection.

**Fig 8:**
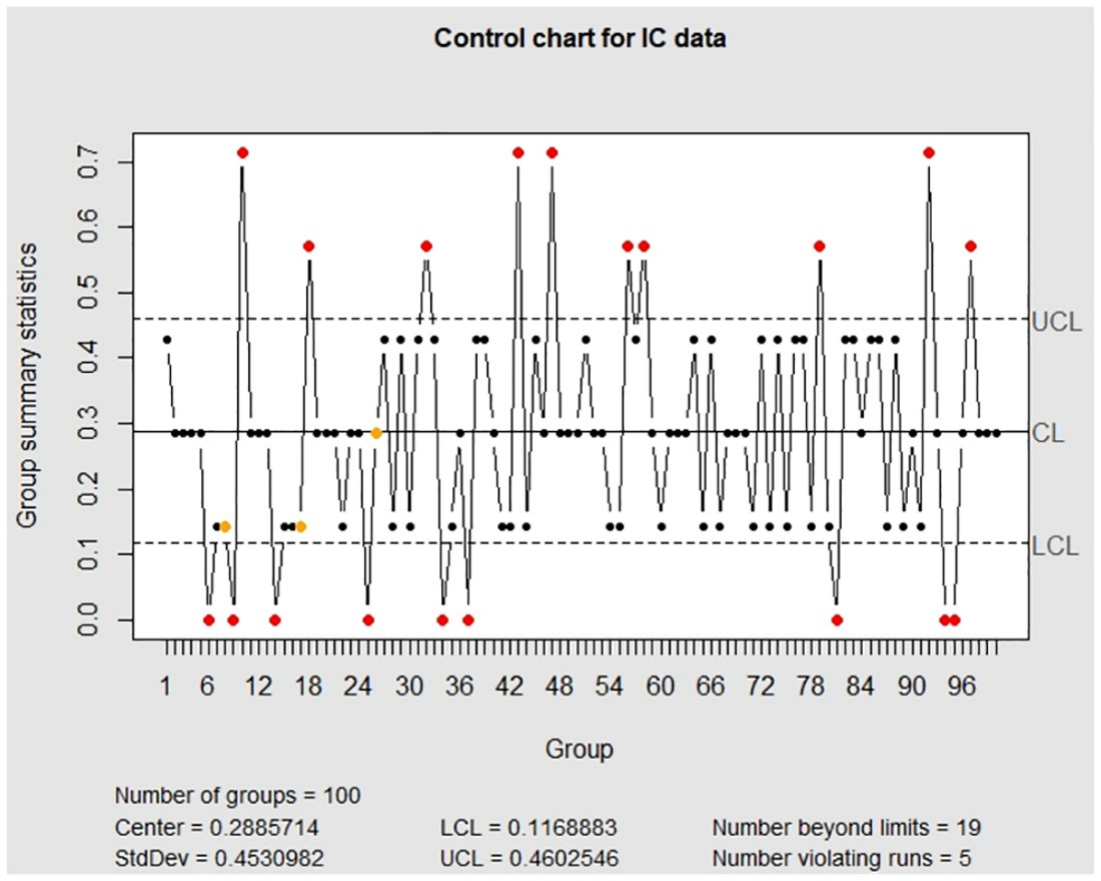
The control charts obtained by the package qcc for the error-prone continuous data. The left panel is for the IC data and the right panel is for the OC data.

Similar to the discussion in Section 5.1, we implement the existing package **qcc** to analyze the error-prone continuous random variables and then compare the performance with our package **EATME**. The resulting control charts are displayed in Fig 8. Similar to the findings in Section 5.1, the trends are fluctuating and there exist several false alarm points in the IC control chart. Moreover, the yellow dots are caused by Western Electric Rules (e.g., [15], p.197). The OC control chart in Fig 8 shows that there are 10 OC data, which is smaller than 14, 11, 15 OC detection in Figs 5–7, respectively. In addition, Table 5 reveals that the package **qcc** produces the smallest LCL and the largest UCL among all numerical results, yielding the widest monitoring region.

**Table 5:** Simulation results and comparisons for the continuous data in Section 5.2. ‘○’ reflects the implementation of truly unobserved data. ‘×’ indicates the implementation of error-prone data without measurement error correction. ‘−’ represents no value. A pair (*x*, *y*) in the column (*π*_11_, *π*_00_) is the implementation of the corrected control chart with parameter values (*π*_11_, *π*_00_) = (*x*, *y*) in (10) accommodated.

Packages	(*π*_11_, *π*_00_)	Output values						
		L1	L2	ARL0	MRL	SDRL	LCL	UCL
**EATME**	°	2.578	2.442	375.107	256	378.674	0.183	0.314
	×	2.563	2.473	361.412	240	377.499	0.210	0.346
	(0.907, 0.924)	2.018	1.994	379.456	257	392.946	0.177	0.306
**qcc**	×	−	−	−	−	−	0.117	0.460

## 6 Real data example

In this section, we implement the package **EATME** to analyze a SECOM dataset that is available on https://archive.ics.uci.edu/ml/datasets/SECOM. This dataset records signals or variables collected under a complex modern semi-conductor manufacturing process. In this dataset, there are 591 features with 1567 in-control samples and 104 out-of-control samples, where a column variable with label -1 reflects IC and 1 represents OC. To demonstrate the idea of the proposed control chart, we take the variable in the second column as an example.

Before analyzing the dataset, we first do preprocessing. Specifically, for the IC sample, we remove missing values and outliers, and obtain the observed data with periods 30 and size 10 in each period. Similarly, we also exclude missing values and outliers for OC samples, yielding the observed data with periods 9 and size 10 in each period.

In the following programming code, we demonstrate the data preparation, where we name the IC and OC samples as IC_SECOM.csv and OC_SECOM.csv, respectively, and two files IC_SECOM.csv and OC_SECOM.csv are available on the GitHub website https://github.com/Kuan-cheng-da/EATME/tree/main.


> library(EATME)
> IC = read.csv('IC_SECOM.csv',header = T)
> OC = read.csv('OC_SECOM.csv',header = T)
> IC_data = matrix(IC[,1],ncol = 10,byrow = T)
> OC_data = matrix(OC[,1],ncol = 10,byrow = T)
> head(IC_data)
> head(OC_data)


Since this dataset contains continuous random variables, we follow our method in Section 2.2 and adopt the function cont_to_disc_V to transform the continuous variable to the discrete one:


> (V = cont_to_disc_V(IC_data,OC_data))
$V0
 [1] 3 1 1 2 1 2 1 1 0 2 1 0 3 2 0 4 2 3 2 1 1 0 2 1 1 1 0 2 2 1

$V1
[1] 1 3 5 4 4 2 2 3 2

$p0
[1] 0.2866667

$p1
[1] 0.5777778

$n
[1] 5


**Fig 9:**
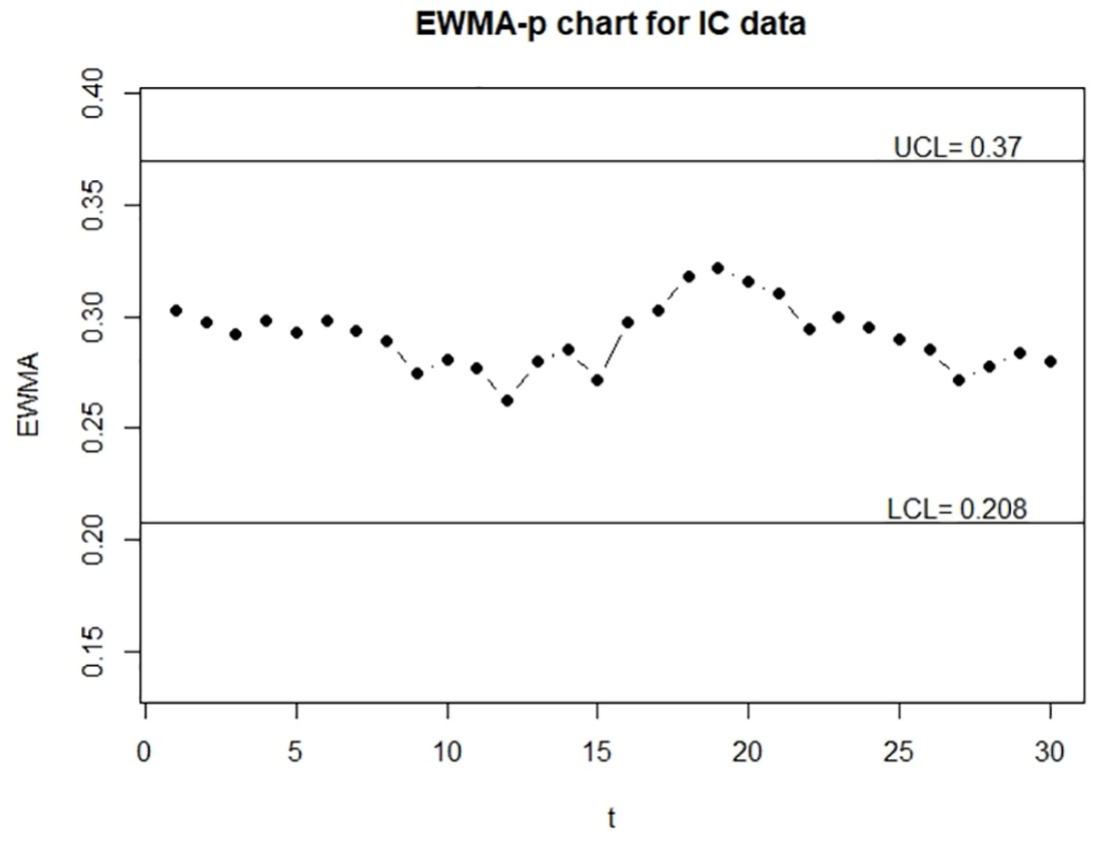
The control charts for error-prone variables in the SECOM data. The left panel is for the IC data and the right panel is for the OC data.


Note that the variable is possibly subject to measurement error as discussed in Section 1. We first implement the variable to construct the two-sided control chart (7) directly, which reflects the scenario that measurement error effects are ignored. The code demonstration, including the estimation of run length, coefficients *L*_3_ and *L*_4_, control limits, and control charts, is given below; and the resulting IC and OC control charts are displayed in Fig 9.

> lambda = 0.05
> (CL0 = EWMA_p_two(V$p0,lambda,V$n,1,1,370.4,3000,10))


*## Draw an IC control chart without measurement error correction*


> EWMA_p_chart_two(V$V0, V$V1, lambda, V$n, 1, 1, 370.4, 3000, 50)


**Fig 10:**
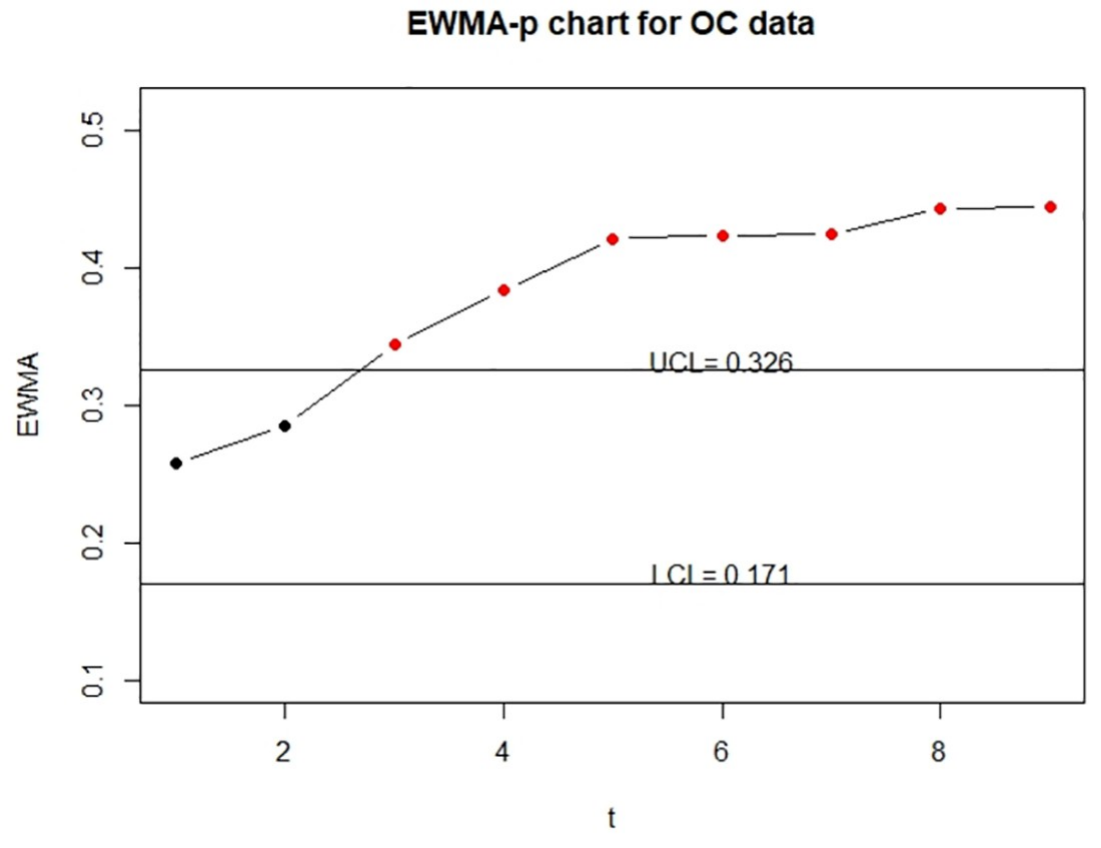
The control charts for error-corrected variables in the SECOM data. The left column is for the IC data and the right column is for the OC data. The first row represents (*π*_11_, *π*_00_) = (0.823, 0.918); The second row is based on (*π*_11_, *π*_00_) = (0.720, 0.870); and the last row is obtained under (*π*_11_, *π*_00_) = (0.616, 0.821).


Next, to deal with measurement error effects, we apply (12) and implement the function EWMA_p_two to construct the corrected EWMA statistic as well as the corresponding control chart. Since *π*_11_ and *π*_00_ are unknown and there is no additional information to estimate them, here we employ sensitivity analyses by specifying several values to *π*_11_ and *π*_00_ and examine the impact of different magnitudes of measurement error effects. In our study, we specify (*π*_11_, *π*_00_) = (0.823, 0.918), (0.720, 0.870), or (0.616, 0.821) and demonstrate the application of programming code below, where the corresponding IC and OC control charts based on different values of *π*_11_ and *π*_00_ are displayed in Fig 10. More detailed demonstration of our code and additional figures can be found on the GitHub: https://github.com/Kuan-cheng-da/EATME/tree/main.

> (CL1 = EWMA_p_two(V$p0,0.05,V$n,0.823,0.918,370.4,3000,10))
> (CL2 = EWMA_p_two(V$p0,lambda,V$n,0.720,0.870,370.4,3000,10))
> (CL3 = EWMA_p_two(V$p0,lambda,V$n,0.616,0.821,370.4,3000,10))


*## Implementation and sensitivity analyses of control charts*

*## with measurement error correction*


> EWMA_p_chart_two(V$V0, V$V1, lambda, V$n, 0.823, 0.918, 
+    370.4, 3000, 50) 
> EWMA_p_chart_two(V$V0, V$V1, lambda, V$n, 0.720, 0.870, 
+    370.4, 3000, 50) 
> EWMA_p_chart_two(V$V0, V$V1, lambda, V$n, 0.616, 0.821, 
+    370.4, 3000, 50)


Compared with charts in Figs 9 and 10, we observe that UCL obtained without measurement error correction is larger than those with measurement error effects addressed. It indicates that the control limit becomes wider if measurement error is ignored. This result directly affects the OC detection. As shown in the right panel of Fig 9, the first OC is detected at *t* = 5 when measurement error is ignored, while the OC in the right panels of Fig 10 is firstly detected at *t* = 3 or 4 if measurement error effects are addressed. It shows that the corrected control chart is sensitive to identify OC.

**Fig 11:**
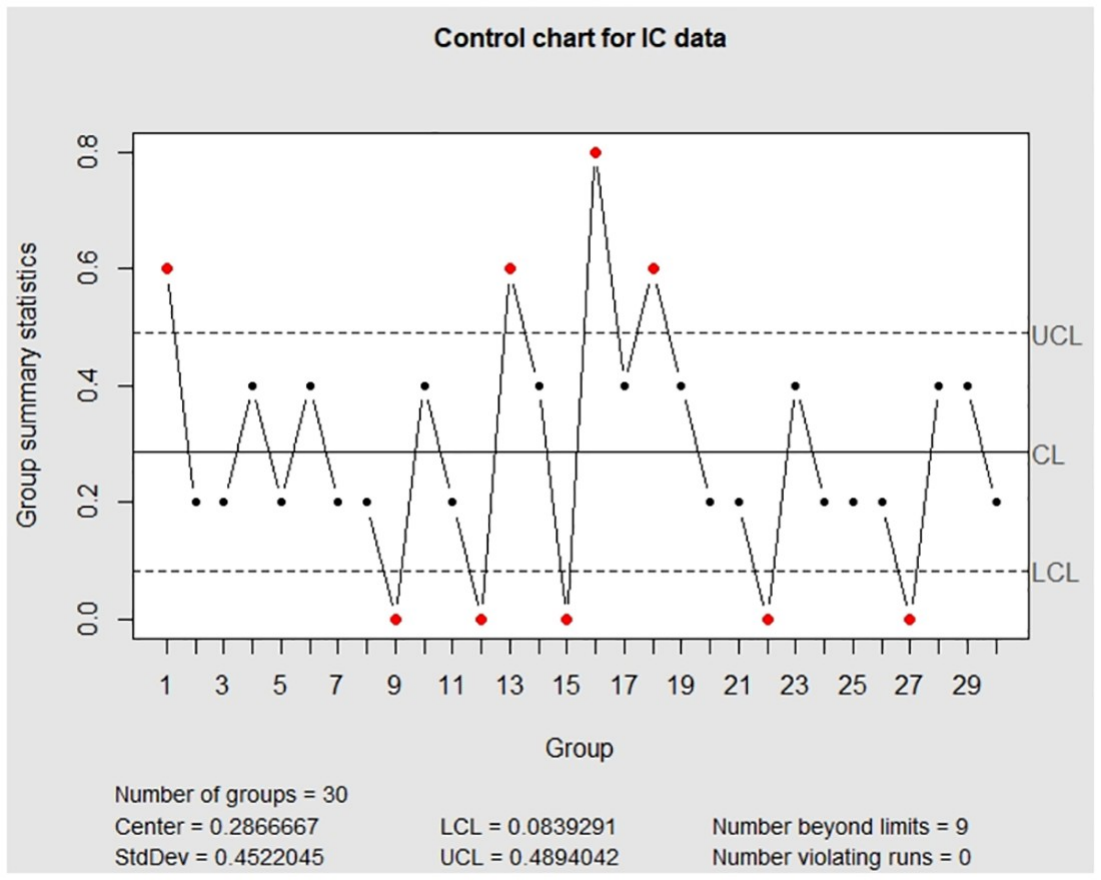
The control charts for error-prone variables in the SECOM data are obtained by the package qcc. The left panel is for the IC data and the right panel is for the OC data.

Finally, to see the impact of measurement error effects and the difference of estimation methods, we implement the variables to the existing method in the package **qcc**, and display the resulting control charts in Fig 11. Similar to the findings in Section 5.1, the package **qcc** produces several false alarm points in the IC control chart. It is interesting to see that the package **qcc** only detects 5 OC points, which is the same as the result in Fig 9, but is less than 6 or 7 OC points in Fig 10. From the summary in Table 6, the monitoring region looks wide if measurement error effects are ignored in the analysis, which is possible to miss OC data. As a result, we can regard this finding as the impact of measurement error effects and the methodology for the monitoring.

**Table 6:** Numerical results and comparisons for the SECOM data in Section 6. ‘×’ indicates the implementation of error-prone data without measurement error correction. ‘−’ represents no value. A pair (*x*, *y*) in the column (*π*_11_, *π*_00_) is the implementation of the corrected control chart with parameter values (*π*_11_, *π*_00_) = (*x*, *y*) in (10) accommodated.

Packages	(*π*_11_, *π*_00_)	Output values						
		*L*_1_	*L*_2_	ARL0	MRL	SDRL	LCL	UCL
**EATME**	×	2.572	2.446	372.352	264	376.03	0.207	0.370
	(0.823, 0.918)	1.655	2.020	377.774	266	384.784	0.198	0.359
	(0.720, 0.870)	1.106	1.783	365.855	254	380.389	0.189	0.347
	(0.616, 0.821)	0.535	1.566	373.969	253	395.845	0.171	0.326
**qcc**	×	−	−	−	−	−	0.084	0.489

## 7 Discussion

In this paper, we introduce a new package called **EATME**, whose primary goal is to correct for measurement error in variables and develop a valid corrected EWMA control chart to monitor defectives when random variables are discrete or explore variance variation when random variables are continuous. In addition, with established control charts, one can further adopt them to detect OC process. This package is useful to handle distribution-free settings and small-sample datasets. The functions in the package give users useful information, including coefficients of control limits and some criteria (e.g., ARL, MRL, and SDRL), to assess the performance of process monitoring and detection. In addition, we also provide plots to clearly show the visualization. Numerical experiments also verify the validity of the corrected EWMA control charts and provide an evidence that ignoring measurement error effects would produce tremendous biases and wrong result.

In the framework of SPC, there are some existing R packages in Table 1 for constructing control charts by various statistics. Unlike those existing packages, the main contributions of the package **EATME** include the correction of measurement error effects and reliable control charts for the detection of the OC data. The arguments in our functions are similar to those in the functions of existing packages, which enable users to input the same dataset to our package and existing R packages, and make comparisons among different packages easy. As commented by a referee, a more convenient implementation of the computation is to integrate the package **EATME** with existing R packages, so that users can make the comparison among different packages and incorporate their existing workflow directly. However, the main challenges in the current status include various purposes (e.g., measurement error problem) and estimation methods (e.g., measurement error correction and derivation for control charts) for packages and data analysis. As a result, the integration of packages is an important but challenging task in the current development, but it is worth exploring in the near future, provided that some issues, such as measurement error correction under different methods in existing packages, can be addressed.
